# Inkjet-printed SnO_x_ as an effective electron transport layer for planar perovskite solar cells and the effect of Cu doping

**DOI:** 10.1098/rsos.231331

**Published:** 2024-02-21

**Authors:** Dongli Lu, Feipeng Yang, Chaochao Dun, Jinghua Guo, Jeffrey J. Urban, Liubov Belova

**Affiliations:** ^1^ Department of Materials Science and Engineering, KTH Royal Institute of Technology, Stockholm 10044, Sweden; ^2^ Joint Center for Energy Storage Research, Lawrence Berkeley National Laboratory, Berkeley, CA 94720, USA; ^3^ The Molecular Foundry, Lawrence Berkeley National Laboratory, Berkeley, CA 94720, USA

**Keywords:** inkjet printing, SnO_x_, Cu doping, perovskite solar cells, hysteresis, low-temperature solution process

## Abstract

Inkjet printing is a more sustainable and scalable fabrication method than spin coating for producing perovskite solar cells (PSCs). Although spin-coated SnO_2_ has been intensively studied as an effective electron transport layer (ETL) for PSCs, inkjet-printed SnO_2_ ETLs have not been widely reported. Here, we fabricated inkjet-printed, solution-processed SnO_x_ ETLs for planar PSCs. A champion efficiency of 17.55% was achieved for the cell using a low-temperature processed SnO_x_ ETL. The low-temperature SnO_x_ exhibited an amorphous structure and outperformed high-temperature crystalline SnO_2_. The improved performance was attributed to enhanced charge extraction and transport and suppressed charge recombination at ETL/perovskite interfaces, which originated from enhanced electrical and optical properties of SnO_x_, improved perovskite film quality, and well-matched energy level alignment between the SnO_x_ ETL and the perovskite layer. Furthermore, SnO_x_ was doped with Cu. Cu doping increased surface oxygen defects and upshifted energy levels of SnO_x_, leading to reduced device performance. A tunable hysteresis was observed for PSCs with Cu-doped SnO_x_ ETLs, decreasing at first and turning into inverted hysteresis afterwards with increasing Cu doping level. This tunable hysteresis was related to the interplay between charge/ion accumulation and recombination at ETL/perovskite interfaces in the case of electron extraction barriers.

## 1. Introduction

Perovskite solar cells (PSCs) have achieved a power conversion efficiency (PCE) reaching 26% after a fast development during the past decade [1]. However, there is still a lack of a reliable large-scale manufacturing method that can reduce the production cost of PSCs and simultaneously offer PSC device performance comparable to that of conventional spin coating. Inkjet printing is considered as an alternative deposition method to spin coating because of its potential for large-scale manufacturing [2]. Moreover, its drop-on-demand technology produces almost no material waste. In recent years, partially inkjet-printed PSCs have been demonstrated [3–5] while inkjet-printed SnO_2_ electron transport layers (ETLs) have not been widely reported. Rohnacher *et al*. fabricated SnO_x_ ETLs using inkjet printing and achieved a PCE of 18.8% with severe hysteresis [6]. Ghahremani *et al*. reported an optimal PCE of 13.08% for devices using inkjet-printed SnO_2_ ETLs [7]. In our previous work, we achieved a PCE of 17.37% for inkjet-printed SnO_2_ ETLs prepared from a commercial colloidal SnO_2_ dispersion [8]. More studies with detailed analysis are required to move closer to large-scale device applications.

As a key component of PSCs, ETLs play an important role in transporting electrons and blocking holes [9]. For a long time, TiO_2_ has served as the most widely used electron transport material because of its abundance and favourable energy band alignment with perovskites [10]. However, TiO_2_ has its own limitations. For example, its photoactivity deteriorates device stability under ultraviolet (UV) illumination; a high sintering temperature (greater than 450°C) is required for proper crystallinity of TiO_2_, which is not compatible with flexible PSCs [11,12]. Thereafter, SnO_2_ has attracted considerable attention because of its potential to overcome the disadvantages of TiO_2_. SnO_2_ has a slightly wider band gap (above 3.6 eV) than TiO_2_, allowing for a longer device stability upon exposure to UV light [13]. It also has an electron mobility of up to 240 cm^2^ V^−1^ s^−1^, which is 100 times higher than that of TiO_2_ [12]. Regarding the fabrication of SnO_2_ ETLs, a high-temperature sintering process is not a must. Low-temperature processed SnO_2_ ETLs have been reported to deliver high device performance [14–16], even superior to that of their high-temperature processed counterparts [17,18]. Up to now, the main strategy for boosting the performance and stability of SnO_2_ based devices has been to suppress energy loss within SnO_2_ [19,20] or at the ETL/perovskite interface [21–23]. Doping is a direct and effective method of modifying the properties of SnO_2_, such as the conductivity and work function, which can facilitate electron extraction and transport [24]. It is also easily compatible with a solution process. For the selection of doping elements, a broad range of dopants has been tested, such as Li [25], Ga [26], Nb [27], Zn [28] and Sb [29]. To the best of our knowledge, there are only two reports in which Cu-doped SnO_2_ was used as an ETL for PSCs. Zhou *et al*. applied Cu-doped SnO_2_ ETLs in planar PSCs and achieved an enhanced PCE of 21% (19.63% for undoped SnO_2_-based PSCs) and improved long-term stability [30]. In the other study [31], based on a low-temperature solution-processed Cu-doped SnO_2_ ETL, the PSC device exhibited a PCE of 11.29% and a fill factor (FF) of 73.38% compared to the undoped device with a PCE of 8.32% and an FF of 59.9%. Therefore, there is a need for more comprehensive studies on the effects of Cu doping on device performance to unveil the underlying mechanisms.

In this study, we conducted a systematic investigation of the application of inkjet-printed solution-processed SnO_x_ ETLs in planar PSCs. The effect of the annealing temperature of SnO_x_ ETLs was studied, and low-temperature amorphous SnO_x_ outperformed high-temperature crystalline SnO_2_. The amorphous SnO_x_ enabled the formation of high-quality perovskite films. SnO_x_ also exhibited improved electrical and optical properties and better energy level alignment with the perovskite layer, facilitating charge transport and reducing charge recombination at ETL/perovskite interfaces. An optimum PCE of 17.55% was achieved for planar PSCs using inkjet-printed amorphous SnO_x_ ETLs. Afterwards, Cu was incorporated into SnO_x_. Upon Cu doping, SnO_x_ exhibited increased surface defects and upshifted energy levels, and the perovskite film exhibited increased defects, resulting in reduced device performance. A tunable hysteresis was observed for PSCs based on Cu-doped SnO_x_ ETLs with variations in the Cu doping level, originating from the interaction of ion/charge accumulation and recombination at ETL/perovskite interfaces in the case of extraction barriers.

## 2. Material and methods

### 2.1. Materials

All chemicals were purchased off the shelf and were used without further modifications. Tin(IV) acetate (Sn(CH_3_CO_2_)_4_) and copper(II) acetate (Cu(CO_2_CH_3_)_2_, 99.99%) were purchased from Sigma–Aldrich (Darmstadt, Germany). Lead iodide (PbI_2_, 99.99%) and lead bromide (PbBr_2_, >98.0%) were purchased from TCI (Tokyo, Japan). Formamidinium iodide (FAI, CH(NH_2_)_2_I, >98%) and methylammonium bromide (MABr, CH_3_NH_3_Br, >98%) were purchased from Dyenamo (Stockholm, Sweden) and Sigma–Aldrich (Darmstadt, Germany), respectively. Spiro-OMeTAD (99.8%) was purchased from Borun New Material Technology (Ningbo, China). Bis(trifluoromethane)sulfonimide lithium salt (LiTFSI, 99.95%), FK 209 Co(III) TFSI salt (FK 209, 98%), and 4-*tert*-butylpyridine (TBP, 98%) were purchased from Sigma–Aldrich (Darmstadt, Germany).

### 2.2. Inkjet printing of pristine and Cu-doped SnO_x_ thin layers

A customized drop-on-demand inkjet printing system was used in our laboratory, which was designed for the piezoelectric-driven printheads from XaarJet [32]. The inkjet printing of SnO_x_ and Cu-doped SnO_x_ was performed under ambient conditions using XJ126/80 printheads with 126 active nozzles and a drop volume of 80 pL. More technical information on the printheads is presented in electronic supplementary material, table S1. A customized waveform was used with a peak voltage of 20 V and a jetting pulse of 20 µs. The printing frequency was set as 283.46 Hz and the printing resolution was 360 dpi.

SnO_x_ thin films were inkjet-printed using inks with tin(IV) acetate dissolved in a solution consisting of 2-propanol and propylene glycol (9/1, v/v). A few drops of ethanolamine were added to improve solubility of tin acetate. For printing, a substrate was placed on a pre-heated printing stage at 60°C; 5 min after printing, the substrate was transferred to a furnace and annealed at 220°C for 1 h. Cu-doped SnO_x_ was fabricated using a process similar to that of pristine SnO_x_. A certain amount of Cu acetate solution, containing 0.25 M Cu acetate dissolved in a mixed solvent of 2-propanol and propylene glycol (9/1, v/v), was added to the Sn acetate solution. The amount of the added Cu acetate solution was determined based on the desired doping level. The mixed solution was then printed, and the resulting film was annealed at 220°C for 1 h.

### 2.3. Device fabrication

Fluorine-doped tin oxide (FTO) glass substrates (14 Ω sq^−1^, Pilkington TEC) were cut into pieces with dimensions of 25 mm × 15 mm. Each piece was etched at the edge with Zn powder and 2 M HCl aqueous solution. After that, the substrates were sonicated in the sequence of 5% deconex water solution, deionized water, acetone, and 2-propanol for 15 min. Before inkjet printing of the ETL, the FTO substrate was pre-heated at 500°C for 30 min (pre-heating treatment) and afterwards cooled down to room temperature. A compact SnO_x_ or Cu-doped SnO_x_ ETL was fabricated as described above. For fabrication of the perovskite layer, we followed the procedure reported in [8,33]. To prepare the perovskite precursor, 1.1 M PbI_2_, 1 M FAI, 0.2 M PbBr_2_ and 0.2 M MABr were dissolved in a mixed solvent (*N*,*N*-dimethylformamide/dimethyl sulfoxide, 4/1 v/v). From that volume, 75 µl of the perovskite precursor was spin-coated at 4500 rpm for 30 s, and meanwhile 125 µl of chlorobenzene was dripped onto the perovskite film 15 s before the end of spin coating. The resulting perovskite film was immediately transferred to a hotplate and dried at 100°C for 30 min. A hole transport layer was prepared by spin-coating at 4000 rpm for 30 s using a precursor containing 70 mM Spiro-OMeTAD, 20 mM LiTFSI, 200 mM TBP, and 2 mM FK 209 in chlorobenzene. Finally, an Au electrode with a thickness of 80 nm was deposited via thermal evaporation (Edwards Auto 306).

### 2.4. Characterization

The surface morphology and cross-sectional microstructure were analysed via a combined focused ion beam/scanning electron microscope (FIB/SEM, FEI Nova 600 Nanolab, ThermoFisher, Eindhoven, Netherlands). The X-ray diffraction (XRD) analysis was performed using an X-ray diffractometer (Siemens D5000, Siemens, Munich, Germany) with the use of Cu K*α*1 radiation (*λ* = 1.5406 Å). The characterized areas of the solar cells were defined by masks of 0.126 cm^2^ and illuminated under an AM 1.5G solar simulator (Newport 91160–1000) with an incident light density of 100 mW cm^−2^. Photocurrent density–voltage (J–V) data were collected by a Keithley 2400 unit with a scan rate of 125 mV s^−1^. X-ray photoelectron spectroscopy (XPS) and ultraviolet photoelectron spectroscopy (UPS) measurements were conducted using a K-Alpha XPS/UPS system manufactured by Thermo Scientific. For XPS analysis, the spectra were obtained using a monochromatized Al K*α* line with a photon energy (*hν*) of 1486.6 eV. For UPS analysis, a He1 ultraviolet light source with an energy of 21.22 eV was employed. The valence band photoelectron signal originated from the top 2–3 nm of the sample surface, and the electronic work function of the material surface was measured. Sn M_5,4_-edge and O K-edge X-ray absorption spectroscopy (XAS) measurements were conducted on BL7.3.1 and 8.0.1.4 at the Advanced Light Source (ALS). The ultraviolet–visible (UV/Vis) absorption and transmission spectra were obtained using a Lambda 750 spectrophotometer. The conductivity of the ETLs was measured using a two-probe method [33], and the current–voltage (I–V) characteristics were collected by a Keithley 2400 unit. The steady-state photoluminescence (PL) of the perovskite films was investigated using a CARY Eclipse fluorescence spectrophotometer with an excitation wavelength of 450 nm.

## 3. Results and discussion

### 3.1. Inkjet-printed solution-processed SnO_x_ electron transport layer

**Figure 1.**
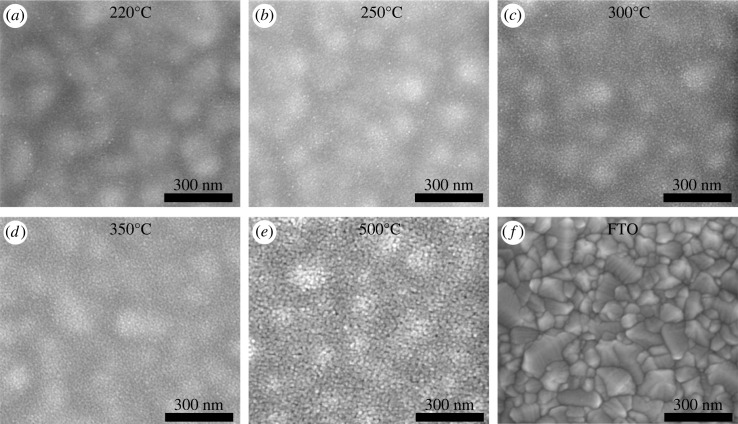
Top-view SEM images of inkjet-printed SnO_x_ thin films annealed at various temperatures: (*a*) 220°C, (*b*) 250°C, (*c*) 300°C, (*d*) 350°C, and (*e*) 500°C. A bare FTO substrate is shown in (*f*). Note that larger spherical features in (*a*–*e*) are from the FTO beneath the SnO_x_ films, which has a substantially higher roughness (*f*).

**Figure 2.**
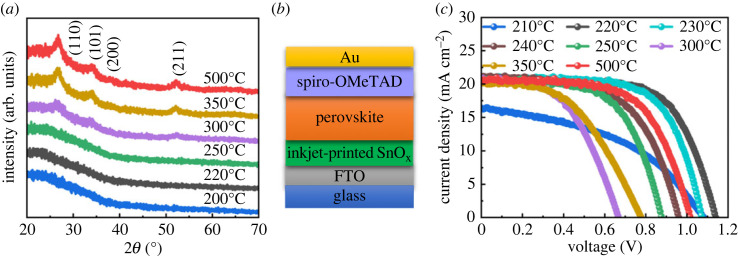
(*a*) Temperature-dependent XRD patterns of SnO_x_ thin films inkjet-printed on glass substrates; (*b*) device configuration and (*c*) J–V curves of PSCs based on inkjet-printed SnO_x_ ETLs annealed at various temperatures.

In this work, we prepared SnO_x_ thin films using precursor inks consisting of tin(IV) acetate. The drying mechanism of these inkjet-printed thin films is different from that of their spin-coated counterparts prepared from SnCl_2_ or tin(IV) isopropoxide precursors [17,18]. Therefore, the effect of the annealing temperature was studied to find the optimal annealing temperature for SnO_x_ ETLs. A schematic diagram of the inkjet-printed solution-processed SnO_x_ is shown in electronic supplementary material, figure S1. Figure 1 shows top-view SEM images of the printed films annealed at various temperatures. All the printed films were uniform and continuous, with no pinholes observed. Obvious granular features appeared at 300°C. The particles became larger and the films exhibited more porosity when the temperature was further increased to 500°C. This was confirmed by the XRD patterns displayed in figure 2*a*. The printed SnO_x_ thin films annealed below 300°C exhibited an amorphous phase with no diffraction peaks detected. The characteristic peaks attributed to tetragonal rutile crystalline phases of SnO_2_ (JCPDS 41-1445) were observed at and above 300°C [17,34]. The intensities of these peaks increased with increasing annealing temperature.

Planar PSCs (figure 2*b*) were then fabricated using inkjet-printed SnO_x_ ETLs annealed at various temperatures ranging from 210°C to 500°C. The J–V curves of the devices are shown in figure 2*c*. The photovoltaic parameters and their statistical distributions are presented in detail in electronic supplementary material, table S2 and figure S2, respectively. The cells based on 220°C-annealed SnO_x_ ETLs achieved the highest PCE of 15.70% (table 1). When the annealing temperature decreased from 220°C to 200°C, the short-circuit current density (J_SC_) and FF both decreased dramatically, and J_SC_ almost dropped to 0 at 200°C. The reason could be that tin acetate did not decompose completely at 200°C, and thus the residuals of the acetate could hinder the charge transfer and act as recombination sites. When the annealing temperature increased from 220°C to 500°C, J_SC_ changed slightly, whereas the open-circuit voltage (V_OC_) and FF varied significantly and showed the same trend, first decreasing and then increasing. Thus, PSCs based on 300°C-annealed SnO_x_ showed the lowest PCE of 7.03%, which could be due to the phase transfer of the resulting SnO_x_ from amorphous to crystalline at 300°C [6]. The PCE then increased to 13.17% when the annealing temperature increased from 300°C to 500°C. Table 1 also demonstrates an important parameter, the hysteresis index (HI). This is used to describe the current–voltage hysteresis and is defined as HI = (PCE|_reverse_ – PCE|_forward_)/PCE|_reverse_, where PCE|_reverse_ and PCE|_forward_ represent the PCE obtained from the reverse-scan and forward-scan J–V characteristics, respectively. PSCs with low-temperature (e.g. 220°C) annealed SnO_x_ ETLs exhibited suppressed hysteresis compared to those with high-temperature (e.g. 500°C) annealed SnO_2_ ETLs.

**Table 1.** Photovoltaic parameters of PSCs with inkjet-printed SnO_x_ ETLs annealed at 220°C and 500°C.

	scan direction	PCE (%)	V_OC_ (V)	J_SC_ (mA cm^−2^)	FF (%)	HI (%)	R_s_ (Ω cm^2^)	R_sh_ (Ω cm^2^)	A	J_0_ (mA cm^−2^)
220°C	reverse	15.70	1.14	20.95	65.7	15.3	3.54	1100	4.03	3.73 × 10^−4^
(SnO_x_)	forward	13.30	1.12	20.82	57.3					
500°C	reverse	13.17	1.02	20.64	62.9	22.5	3.90	650	4.15	1.56 × 10^−3^
(HT-SnO_2_)	forward	10.21	0.91	20.81	53.9					

The results presented above suggest that the low-temperature annealed SnO_x_ ETL outperformed its high-temperature counterpart, which are consistent with reported results [17,18]. We then conducted additional experiments and characterizations to reveal the origins of the improved performance of the low-temperature annealed SnO_x_ ETLs. For simplicity, further on we only discuss the results for the low-temperature (220°C) annealed SnO_x_ and the high-temperature (500°C) annealed SnO_2_ (HT-SnO_2_).

**Figure 3.**
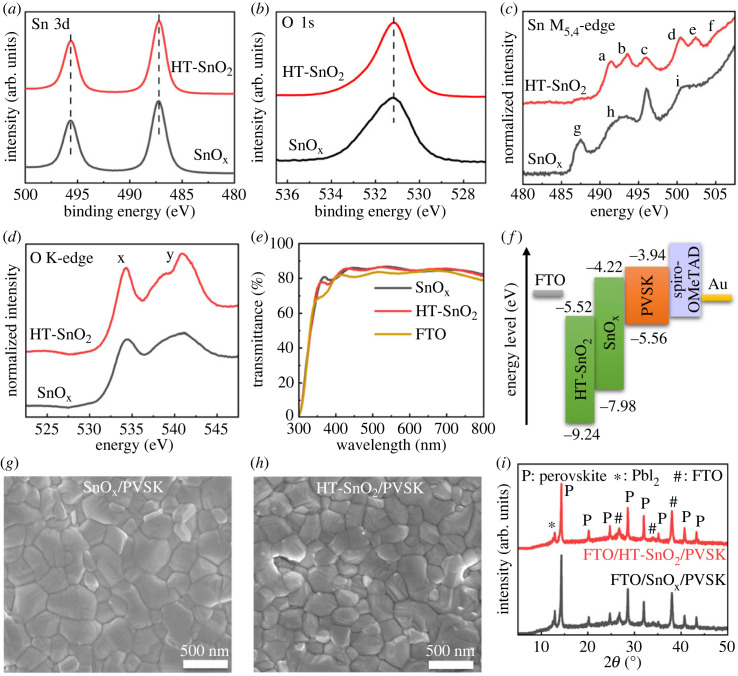
(*a*) Sn 3d XPS, (*b*) O 1s XPS, (*c*) Sn M_5,4_-edge XAS, (*d*) O K-edge XAS, and (*e*) optical transmission spectra of SnO_x_ and HT-SnO_2_; (*f*) energy level alignment of the PSC device with a SnO_x_ or HT-SnO_2_ ETL; (*g*,*h*) SEM images and (*i*) XRD patterns of the perovskite films deposited on SnO_x_ and HT-SnO_2_.

XPS measurements were conducted to investigate the chemical states of SnO_x_ and HT-SnO_2_. As seen in figure 3*a*, SnO_x_ showed two characteristic peaks at 495.7 eV and 487.3 eV (ΔE = 8.4 eV), which were assigned to Sn 3d_3/2_ and Sn 3d_5/2_, respectively. SnO_x_ also exhibited a peak at 931.2 eV in the O 1s XPS spectrum (figure 3*b*). These XPS results suggest that Sn was in the +4 valence state in SnO_x_. There was no peak shift observed between SnO_x_ and HT-SnO_2_ in Sn 3d and O 1s XPS spectra, which indicates that Sn was also in the +4 state in HT-SnO_2_. Moreover, XAS measurements were performed to confirm the amorphous nature of SnO_x_. In the Sn M_5,4_-edge XAS spectra of HT-SnO_2_ (figure 3*c*), two sets of triplet peaks (a, b, c) and (d, e, f) arose from 3d_5/2_ and 3d_3/2_ to 5p transitions, respectively. In figure 3*d*, peaks x and y corresponded to the hybridization of O 2p orbitals with Sn 5s and Sn 5p orbitals, respectively [35]. The Sn M_5,4_-edge and O K-edge spectral features of HT-SnO_2_ are consistent with the typical characteristics of crystalline SnO_2_ in earlier reports [34,36,37]. For the SnO_x_ sample, significant differences were observed in the Sn M_5,4_-edge spectral features. Peaks a and b broadened and merged into one peak h, and peaks d and e broadened and merged into i as well. This spectral broadening is related to the lack of long-range order in the structure of SnO_x_ and the presence of uncoordinated surface tin atoms [38,39]. An additional pre-edge peak g appeared, which was assigned to the surface states originating from uncoordinated surface atoms, oxygen vacancies, or surface reconstruction [38–40]. As observed in the O K-edge spectrum of SnO_x_, the spectral broadening was consistent with that in the Sn M_5,4_-edge spectrum and was attributed to the presence of the surface states. These Sn M_5,4_-edge and O K-edge spectral features of SnO_x_ are consistent with those of the amorphous SnO_x_ reported previously [37,41,42], confirming an amorphous structure without long-range order for the low-temperature annealed SnO_x_.

The amorphous structure of SnO_x_ can be beneficial for the charge transport within ETLs. The grain boundary scattering within the nanocrystalline HT-SnO_2_ could lead to electron transport losses, which would be less severe in the amorphous SnO_x_ [43,44]. This was verified by the improved conductivity of SnO_x_ (electronic supplementary material, figure S3). The conductivity of SnO_x_ was estimated to be 2.96 × 10^−5^ S cm^−1^, which was slightly higher than that of HT-SnO_2_ (2.46 × 10^−5^ S cm^−1^). This enhanced conductivity can contribute to the improved J_SC_. UV/Vis measurements were performed to examine the optical properties of SnO_x_ and HT-SnO_2_ (figure 3*e*). SnO_x_ and HT-SnO_2_ both showed an optical transmittance above 80% in the visible region (380–750 nm), which confirmed the transparency of the two samples. They also improved the optical transmission properties of FTO substrates because of the good antireflection ability of SnO_2_ thin films [44,45]. Compared to HT-SnO_2_, SnO_x_ exhibited a slightly enhanced transmittance, which could allow more light to reach the perovskite absorber to generate carriers, contributing to the enhanced J_SC_ of the devices based on SnO_x_ ETLs [45]. Furthermore, the optical band gap (E_g_) can be determined by Tauc's relationship, which is described by ${\left(\alpha h\nu \right)}^{2}=C\times (h\nu -E_{g})$, where *α* is the optical absorption coefficient, *hν* is the photon energy, and *C* is a material constant [46]. The values of the band gap extrapolated from Tauc's plots (electronic supplementary material, figure S4) were 3.76 eV and 3.72 eV for SnO_x_ and HT-SnO_2_, respectively. The enlarged band gap of SnO_x_ was consistent with the enhanced transmittance.

UPS measurements were performed to investigate the energy band levels of SnO_x_ and HT-SnO_2_ (electronic supplementary material, figure S5*a*). The work function can be determined by the formula $E_{F}=21.22-(E_{\mathrm{onset}}-E_{\mathrm{cutoff}})$ [22], where *E*_F_ is the Fermi level, *E*_onset_ is the onset, and *E*_cutoff_ is the cutoff of the UPS spectrum. The work function of SnO_x_ and HT-SnO_2_ was calculated to be 6.76 eV and 7.02 eV, respectively. The valence band maximum (E_VBM_) of SnO_x_ and HT-SnO_2_ was measured to be 1.22 eV and 2.22 eV below the Fermi level (electronic supplementary material, figure S5*b*), and then the E_VBM_ values were calculated to be −7.98 eV and −9.24 eV, respectively. Their conduction band minimums (E_CBM_) were further determined to be −4.22 eV and −5.52 eV using the equation $E_{\mathrm{CBM}}=E_{\mathrm{VBM}}+E_{g}$. The energy band positions of the perovskite layer were obtained from the literature [47]. The energy band alignment diagram for PSCs based on SnO_x_ and HT-SnO_2_ ETLs is shown in figure 3*f*. Compared to HT-SnO_2_, SnO_x_ showed upshifted energy band levels and thus possessed an E_CBM_ much closer to that of the perovskite layer, enabling more efficient electron extraction and less V_OC_ losses at the SnO_x_/perovskite interfaces. Moreover, the E_CBM_ of HT-SnO_2_ was very close to the E_VBM_ of the perovskite layer, which could result in severe recombination of the electron–hole pairs at the HT-SnO_2_/perovskite interfaces [48]. Therefore, SnO_x_ exhibited a better energy level alignment with the perovskite layer, thereby facilitating electron extraction and reducing recombination losses at the SnO_x_/perovskite interfaces [49,50]. This could be the main reason why SnO_x_ ETLs offered improved V_OC_ and suppressed hysteresis compared with HT-SnO_2_ ETLs.

The film morphology of the perovskite layers deposited on SnO_x_ and HT-SnO_2_ was also investigated. As shown in the SEM images (figure 3*g*,*h*), the perovskite film deposited on SnO_x_ exhibited a larger average grain size of 293 nm than that of 242 nm for the perovskite film deposited on HT-SnO_2_ (electronic supplementary material, figure S6). A few gaps appeared for the perovskite film deposited on HT-SnO_2_. In the XRD patterns of the two perovskite films illustrated in figure 3*i*, three major peaks located at 14.3°, 28.6°, and 32.0° were assigned to (001), (002), and (012) crystal planes, respectively [51,52]. Compared to the HT-SnO_2_/perovskite film, the SnO_x_/perovskite film displayed increased relative intensity at both (001) and (002) peaks (electronic supplementary material, table S3), indicating enhanced crystallinity of the perovskite film with preferential growth along (001) and (002) planes [52,53]. Overall, the data showed that the SnO_x_/perovskite film exhibited enlarged grains, improved morphology, and enhanced crystallinity. This might be attributed to the improved morphology of the amorphous SnO_x_, thereby boosting the formation and crystallization of the perovskite film. The improved film quality of the perovskite layer can facilitate charge transport and reduce carrier recombination at grain boundaries, leading to enhanced device performance [54]. PL measurements were performed to investigate the charge transport kinetics at ETL/perovskite interfaces (electronic supplementary material, figure S7). The faster quenching indicated more efficient charge extraction and transport at the SnO_x_/perovskite interface. Furthermore, the recombination characteristics of PSCs with SnO_x_ and HT-SnO_2_ ETLs were obtained by fitting their J–V curves using an ideal diode mode: $J=J_{\mathrm{ph}}-J_{0}\times \exp[e(V+J\times R_{s})/AKT]$ (electronic supplementary material, figure S8), where *J*_ph_ is the photo-induced current density, *J*_0_ is the recombination current density, *e* is the elementary charge, *R*_s_ is the series resistance, *A* is the ideality factor, *K* is the Boltzmann constant, and *T* is the thermodynamic temperature [55]. The shunt resistance (*R*_sh_) is calculated as $R_{\mathrm{sh}}=-dV/dJ$ at $J=J_{SC}$. The values of these parameters are summarized in table 1. J_0_ and the ideality factor both decreased, indicating suppressed charge recombination in the cells with SnO_x_ ETLs [45,55]. The reduced *R*_s_ and increased *R*_sh_ was also indicative of effective electron extraction and suppressed charge recombination at SnO_x_/perovskite interfaces [43]. To summarize, the enhanced charge extraction and transport and suppressed charge recombination were ascribed to the enhanced electrical and optical properties of the amorphous SnO_x_, improved film quality of the perovskite layer, and good energy level matching between the SnO_x_ ETL and the perovskite layer. Consequently, PSCs with SnO_x_ ETLs achieved improvements in all photovoltaic parameters (V_OC_, J_SC_, FF and PCE) and suppression of hysteresis compared to those with HT-SnO_2_ ETLs.

**Figure 4.**
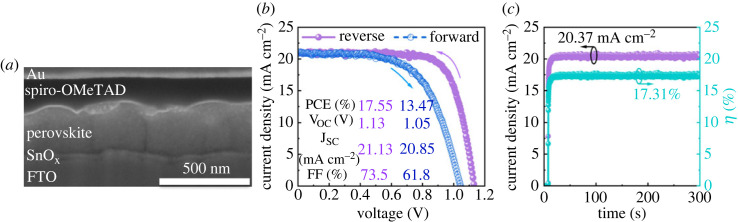
(*a*) Cross-sectional FIB/SEM image, (*b*) reverse-scan and forward-scan J–V curves, and (*c*) steady-state photocurrent density and stabilized efficiency (at a bias of 0.85 V) for the champion cell based on the inkjet-printed SnO_x_ ETL prepared from a 0.05 M precursor ink.

The surface wettability of the substrates is crucial to film formation in the inkjet-printing deposition process, especially in a low-temperature solution process. Specifically, we fabricated three groups of PSCs using SnO_x_ ETLs printed on FTO substrates without surface treatment, with ultraviolet/ozone (UVO) treatment, and with pre-heating treatment. As shown in electronic supplementary material, figure S9 and table S4, the device performance was improved by applying either UVO or pre-heating treatment. Both UVO and pre-heating treatment can remove the organic residuals on the FTO substrates and improve the wettability of the FTO substrates. This enabled the uniform deposition of SnO_x_ on the FTO substrates (electronic supplementary material, figure S9) and improved the interfacial contact between SnO_x_ and FTO, contributing to reduced recombination and thus improved performance with suppressed hysteresis [56,57]. Therefore, pre-heating treatment is a simple alternative to UVO cleaning in terms of substrate surface processing. We further optimized the performance of SnO_x_-based devices by controlling the thickness of the SnO_x_ ETL, which was realized by adjusting the concentration of the precursor inks. As shown in figure 4*a* and electronic supplementary material, figure S10, the average thickness of the SnO_x_ ETL increased from 0 to 35 nm and 75 nm when the precursor concentration increased from 0 to 0.05 M and 0.1 M. Accordingly, the device performance varied with the thickness variation of the SnO_x_ ETL (electronic supplementary material, figure S11, figure S12, and table S5). The highest PCE of 17.55%, along with a forward-scan PCE of 13.47% (figure 4*b*), was obtained for the cell based on a SnO_x_ ETL with a thickness of approximately 35 nm. We also obtained a stabilized photocurrent density of 20.37 mA cm^−2^ and stabilized power output of 17.31% over 5 min (figure 4*c*). Moreover, PSCs with SnO_x_ ETLs exhibited improved performance with suppressed hysteresis compared with those without an ETL, regardless of the thickness of the SnO_x_ ETL. This proved that the inkjet-printed SnO_x_ as an ETL had the capability of separating charges, i.e. transporting electrons and blocking holes [17].

In comparable reports focused on inkjet-printed SnO_2_ ETLs, Ghahremani *et al*. achieved a champion PCE of 13.08% [7], and Rohnacher *et al*. obtained a champion efficiency of 18.8% (12.0% in the forward scan) with a stabilized PCE of 15.2% over 5 min [6]. Our devices exhibited an improved stabilized efficiency and suppressed hysteresis compared to those in the latter report, although they did not deliver a PCE higher than 18.8%. Overall, we successfully fabricated solution-processed SnO_x_ ETLs as effective ETLs for planar PSCs by inkjet printing, which is expected to promote large-scale fabrication of PSCs.

### 3.2. Inkjet-printed solution-processed Cu : SnO_x_ electron transport layer ETL

**Figure 5.**
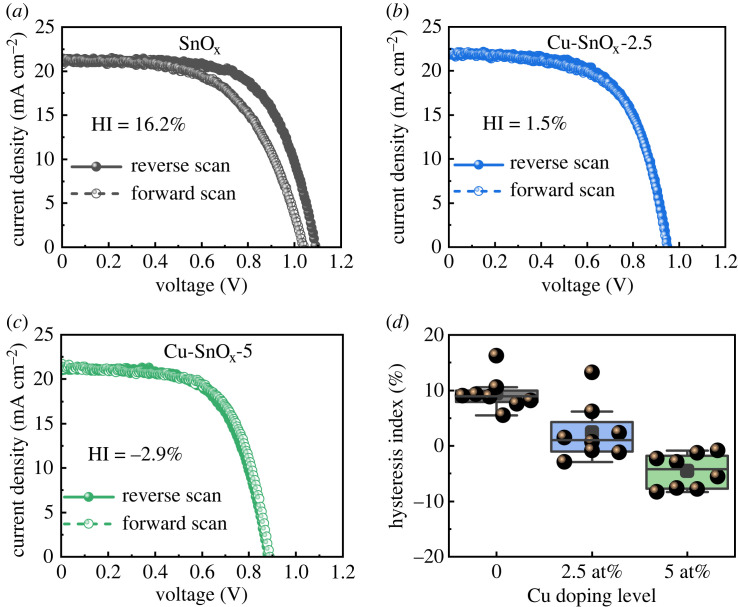
Reverse-scan and forward-scan J–V characteristics of PSCs based on (*a*) pristine SnO_x_, (*b*) 2.5 at% Cu : SnO_x_, and (*c*) 5 at% Cu : SnO_x_; (*d*) distribution of the hysteresis index for PSCs based on pristine SnO_x_ and Cu : SnO_x_ ETLs.

Inkjet-printed solution-processed SnO_x_ has been established as an effective ETL for planar PSCs while there is still a motivation to further optimize this SnO_x_ ETL. The solution process used in the present work allows for doping SnO_x_ by adding a dopant precursor directly into the Sn precursor inks. Herein, we introduced Cu dopant into SnO_x_ ETLs and investigated the effects of Cu doping on the device performance. Various Cu : SnO_x_ ETLs were prepared from inks with different Cu concentrations of 2.5 at%, 5 at%, 7.5 at% and 10 at%. We then fabricated PSCs employing these Cu : SnO_x_ ETLs and obtained J–V characteristics of the devices (figure 5 and electronic supplementary material, figure S13). V_OC_ and FF decreased after Cu doping, while J_SC_ first increased and then decreased with increasing Cu doping level (electronic supplementary material, figure S14 and table S6). As a result, the devices with Cu : SnO_x_ ETLs produced lower PCEs than those with pristine SnO_x_ ETLs. Interestingly, the hysteresis index decreased dramatically from 16.2% to 1.5% when the Cu concentration increased from 0 to 2.5 at%. Afterwards, an inverted hysteresis phenomenon appeared, i.e. the PCE for the forward scan surpassed that for the reverse scan, when the Cu doping level further increased to 5 at% and higher levels. The inverted hysteresis mainly originated from the improved FF for the forward scan compared to that for the reverse scan (electronic supplementary material, figure S14). In addition, we fabricated PSCs using 500°C-annealed Cu : SnO_x_ ETLs. The devices exhibited reduced performance compared to those with 220°C-annealed counterparts (electronic supplementary material, figure S15). This result is consistent with that for the undoped SnO_x_.

**Figure 6.**
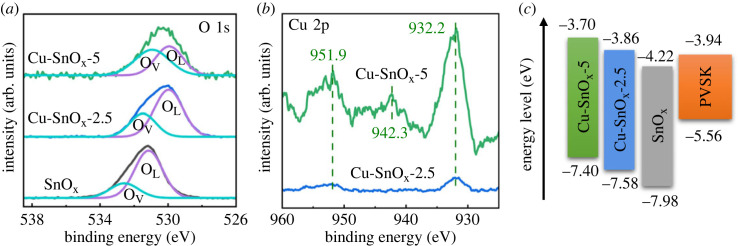
(*a*) O 1s and (*b*) Cu 2p XPS spectra for pristine, 2.5 at%, and 5 at% Cu-doped SnO_x_; (*c*) energy level alignment of the pristine, 2.5 at%, and 5 at% Cu-doped SnO_x_ ETL with the perovskite layer.

To reveal the origin of the effects of Cu doping on the device performance, various characterizations and measurements were conducted. The XRD patterns in electronic supplementary material, figure S16, show that no crystalline peaks were observed for Cu : SnO_x_. All the Cu : SnO_x_ thin films exhibited dense and compact surfaces with no pinholes (electronic supplementary material, figure S17). The SEM images also imply that Cu doping did not have a significant influence on the film morphology. From figure 6*a* and electronic supplementary material, figure S18, the peaks of the O 1s and Sn 3d XPS spectra shifted to lower binding energies upon Cu doping, indicating a strong chemical interaction between Cu and SnO_x_ [26]. As seen in figure 6*b*, the Cu 2p peaks were detected for 5 at% Cu : SnO_x_ while the signal was very weak for 2.5 at% Cu : SnO_x_. The energy peak at 932.2 eV indicated the presence of Cu^+^ oxidation state, and the observable satellite features at 942.3 eV represented the +2 state of Cu [30]. Therefore, there were two oxidation states of Cu^+^ and Cu^2+^ in Cu : SnO_x_. Moreover, both Sn M_5,4_-edge and O K-edge XAS spectra only exhibited intensity variations in the characteristic peaks upon Cu doping (electronic supplementary material, figure S19). The XPS and XAS results suggested that SnO_x_ was successfully doped with Cu. As shown in figure 6*a*, the O 1s XPS peak of SnO_x_ can be deconvoluted into two peaks. The peak at 531.2 eV originated from lattice oxygen (O_L_) and the other peak at 532.6 eV was assigned to oxygen vacancies or adsorbed hydroxyl groups (O_V_) [58,59]. The peak ratio of O_V_ to total oxygen increased from 24.11% to 30.92% when the Cu doping concentration increased from 0 to 2.5 at%, and then significantly increased to 52.53% when the Cu doping level increased to 5 at% (electronic supplementary material, table S7). These surface oxygen defects (oxygen vacancies or hydroxyl groups) could cause carrier recombination and poor charge transfer for PSCs [30,60,61].

The surface chemistry of ETLs can influence the formation and crystallinity of the perovskite layers [30]. For the perovskite film deposited on Cu-doped SnO_x_ compared to that deposited on SnO_x_ (electronic supplementary material, figure S20, and figure 3*g*), the average crystal grain size decreased, and a few white PbI_2_ spots appeared. This was confirmed by the XRD patterns (electronic supplementary material, figure S21), in which the intensity ratio of the peak at 14.3° for perovskite to the peak at 12.9° for PbI_2_ decreased upon Cu doping (electronic supplementary material, table S8). Thus, Cu-doped SnO_x_ exerted a negative effect on the film quality of the perovskite layers, resulting in recombination losses and poor charge transport. This effect was further investigated by PL measurements of the perovskite layers (electronic supplementary material, figure S22). The higher PL intensity represented more severe charge recombination and worse charge transport ability at the Cu-doped SnO_x_/perovskite interfaces [26,31]. The Cu-SnO_x_-5/perovskite film exhibited a much higher PL intensity than the Cu-SnO_x_-2.5/perovskite film, which might be mainly attributed to the presence of a substantial number of oxygen defects on the surface of the 5 at% Cu-doped SnO_x_. Consequently, PSCs based on Cu-doped SnO_x_ ETLs suffered from degradation in device performance (V_OC_, FF, and PCE). With a few exceptions, the devices with 2.5 at% Cu-doped SnO_x_ offered an enhanced J_SC_ compared to those with pristine SnO_x_, which was attributed to the improved conductivity (electronic supplementary material, figure S23) [25].

The optical transmittance of the Cu-doped samples decreased slightly compared to that of the pristine SnO_x_ (electronic supplementary material, figure S24*a*). Then, the values of the optical band gap extracted from Tauc's plots (electronic supplementary material, figure S24*b*) were 3.72 eV and 3.70 eV for 2.5 at% and 5 at% Cu-doped SnO_x_, respectively. The reduced band gap could originate from the increased surface defect states upon Cu doping [62]. Furthermore, the energy band positions of Cu-doped SnO_x_ were obtained from the UPS and valence band XPS measurements (electronic supplementary material, figure S25 and table S9). As shown in figure 6*c*, the conduction band minimum of the two Cu-doped SnO_x_ ETLs was significantly upshifted to become shallower than that of the perovskite layer, which could form unfavourable electron extraction barriers and thus cause charge/ion accumulation at the ETL/perovskite interface [63]. The conduction band offset between Cu-doped SnO_x_ and perovskite increased when the Cu doping level increased from 2.5 at% to 5 at%, leading to increased charge/ion accumulation at the ETL/perovskite interface [64,65]. This could account for the increase in HI (absolute value). The decrease in HI from 0 to 2.5 at% was probably due to the enhanced conductivity which might alleviate the hysteresis by balancing the electron flux and hole flux with PSCs [25,66].

The origin of normal hysteresis has been attributed to ion migration and accumulation at interfaces, which screens the electric field and leads to a decrease in V_OC_ and FF in the forward scan [67–69]. According to a previous study [70], in the case of extraction barriers, these accumulated ionic charges also formed dipole layers with piled-up electronic charges at the ETL/perovskite interface, which enhanced the electric field and improved the FF for the forward scan, resulting in an inverted hysteresis. Therefore, the observed inverted hysteresis in our devices was related to the charge/ion accumulation induced by the energetic extraction barriers at Cu-doped SnO_x_/perovskite interfaces. Besides, the relatively low scan rate (125 mV s^−1^) used in this work also allowed the occurrence of a slow ion migration process, thus intensifying the impact of extraction barriers on charge extraction, leading to an inverted hysteresis [65,70]. Furthermore, the hysteretic behaviour transferred from normal hysteresis to inverted hysteresis when the Cu doping level increased from 2.5 at% to 5 at%, although there were energy barriers at both Cu-doped SnO_x_/perovskite interfaces. A similar phenomenon was reported by Rong *et al*., who achieved tunable hysteresis for carbon-based PSCs by adjusting the thickness of a compact TiO_2_ ETL [71]. They ascribed the origin of this phenomenon to the interplay between the slow dynamics of charge accumulation and changes in recombination rates. In our work, in addition to the changes in charge/ion accumulation induced by the enlarged conduction band offset, the recombination losses at the ETL/perovskite interfaces also varied, originating from variations in the quantity of oxygen defects on the Cu-doped SnO_x_ surfaces and defects on the perovskite films with increasing Cu content. Therefore, the transformation of hysteresis from normal to inverted can be attributed to the interplay between charge/ion accumulation and recombination at the Cu-doped SnO_x_/perovskite interfaces. The exact origin of this phenomenon is still under investigation.

Our results regarding the effect of Cu doping were quite different from those reported in the literature [30,31], in which Cu-doped SnO_2_ based PSCs exhibited an improved PCE with normal hysteresis. The disparity in the device performance could originate from the differences in the morphological, structural, electronic, and optical properties between our Cu-doped SnO_x_ and their Cu-doped SnO_2_. Our results imply that elemental doping does not always lead to a favourable performance improvement. The selection of the doping element and the concentration of the selected dopant are of great importance in determining the PSC device performance. This work demonstrates a method for tuning the hysteresis of SnO_x_-based PSCs through Cu doping. An acceptable PCE of 13.15% with a low hysteresis of 1.5% was obtained for the device with a 2.5 at% Cu-doped SnO_x_ ETL. However, further investigations including stability measurements are required to verify the advantages of this low hysteresis.

## 4. Conclusion

In summary, we have successfully fabricated solution-processed inkjet-printed SnO_x_ as effective ETLs for planar PSCs. The effect of the annealing temperature of the SnO_x_ ETL on the device performance was studied. The low-temperature annealed amorphous SnO_x_ ETLs outperformed their high-temperature annealed crystalline counterparts. The improved device performance originated from enhanced charge extraction and transport and suppressed charge recombination at ETL/perovskite interfaces, which was attributed to the enhanced electrical and optical properties of the amorphous SnO_x_, improved film quality of the perovskite layer, and better energy level alignment between the SnO_x_ ETL and perovskite layer. Through further optimization of the substrate surface treatment and the film thickness of the SnO_x_ ETL, a champion efficiency of 17.55% was achieved. Thereafter, Cu was incorporated into SnO_x_. Upon Cu doping, SnO_x_ showed increased surface defects and upshifted energy levels, and the perovskite film also exhibited increased defects, resulting in the reduction in the device performance. Moreover, the devices with Cu-doped SnO_x_ ETLs exhibited tunable hysteresis, which was suppressed and then transformed into an inverted hysteresis with increasing Cu doping concentration. The reduced hysteresis for the 2.5 at% Cu-doped SnO_x_ ETLs was probably due to the enhanced conductivity. The inverted hysteresis was related to the energetic extraction barriers at Cu-doped SnO_x_/perovskite interfaces. Therefore, the transformation of hysteresis from normal to inverted can be attributed to the interplay between charge/ion accumulation and recombination at Cu-doped SnO_x_/perovskite interfaces in the case of extraction barriers. This work provides an approach to fabricate ETLs in a sustainable and scalable manner and a platform for the fabrication of fully inkjet-printed PSCs in future work.

## Data Availability

The datasets supporting this article have been uploaded as part of the electronic supplementary material [72].
